# Isolation and characterization of novel primary cells from the human distal outflow pathway

**DOI:** 10.1038/s41598-021-83558-6

**Published:** 2021-02-17

**Authors:** Uttio Roy Chowdhury, Cindy K. Bahler, Cheryl R. Hann, Bradley H. Holman, Michael P. Fautsch

**Affiliations:** Department of Ophthalmology, Mayo Clinic, 200 First Street SW, Rochester, MN 55905 USA

**Keywords:** Biological techniques, Cell biology, Molecular biology

## Abstract

Ocular hypertension occurs due to increased resistance to aqueous humor removal through the conventional outflow pathway. Unlike the proximal region of the conventional outflow pathway, the distal region has not been well studied, mostly due to lack of model systems. Here we describe isolation and characterization of human primary vascular distal outflow pathway (VDOP) cells from the distal region of the conventional outflow pathway. Tissue from the distal region was isolated from human corneo-scleral rims, digested with collagenase type I (100 U/ml) and placed on gelatin coated plates to allow cellular growth in Dulbecco’s Modified Eagle’s Medium (low glucose) containing fetal bovine serum and antibiotic/antimycotic. VDOP cells showed consistent proliferation for up to 7 passages, retained endothelial-like nature of the parent tissues and showed a unique marker phenotype of Lectin^+^VEGFR2^-^CD34^-^NG2^-^ that was distinct from neighboring trabecular meshwork (Lectin^+^VEGFR2^-^CD34^-^NG2^+^) and Schlemm’s canal (Lectin^+^VEGFR2^+^CD34^+^NG2^+^) cells. Dexamethasone treated VDOP cells did not express myocilin and did not form cross-linked actin networks, in contrast to trabecular meshwork cells. These data show that VDOP cells are unique to the distal outflow region and can be used as a viable in vitro model system to understand the biology of the distal outflow pathway and intraocular pressure regulation.

## Introduction

Aqueous humor, the primary fluid nourishing the avascular tissues of the anterior chamber of eyes, is produced from the epithelium of the ciliary body and exits the anterior chamber through one of two pathways. The conventional (trabecular) pathway, comprising the trabecular meshwork (TM), Schlemm’s canal (SC), collector channels and aqueous veins, is the primary pressure-dependent pathway for removing aqueous humor in humans. A secondary route called the uveoscleral outflow pathway is pressure-independent where aqueous humor is removed through the interstitial spaces of the ciliary muscles^1^. A major portion of intraocular pressure in the anterior chamber is generated due to homeostatic resistance to aqueous humor passage at the interface between the proximal region of the TM and inner wall of SC^1,2^. Reduction of aqueous humor flow across the TM and SC interface is recognized as the primary cause of elevated intraocular pressure in ocular hypertensive diseases like glaucoma^3–7^.

While the complete molecular events leading to pathological changes in outflow resistance is not understood, there is a wealth of data from in vitro cell culture models of primary normal trabecular meshwork (NTM) and SC cells that have helped describe cellular and molecular events that are altered in normal and ocular hypertensive conditions. For example, primary NTM cells, initially described by Polansky et al. in 1979, have been utilized to characterize their unique properties such as phagocytosis, expression and upregulation of myocilin in response to glucocorticoids, and development of cross-linked actin networks (CLANs)^8–13^. Similarly, establishment of primary cultures of SC cells^14^ have helped understand how changes in physical characteristics of these cells (e.g. stiffness) contribute towards increased resistance to fluid flow^15^.

In addition to outflow resistance generated at the TM and SC interface, evidence also suggests that the distal region of the conventional outflow pathway (region immediately downstream to the outer wall of SC comprising the deep scleral, intrascleral and episcleral plexus regions, referred to as the distal outflow pathway in this article) has a significant role in regulating outflow resistance^16–18^. Even after complete removal of the TM area, human eyes can still retain up to 50% resistance to outflow^17–23^. Surgical techniques that remove parts of the distal outflow pathway while maintaining the integrity of the TM, cause similar increases to outflow facility as with removal of TM alone^24–27^. The pulsatile flow of aqueous humor in the distal outflow pathway is lost during glaucoma with a direct correlation between the loss of pulsatile flow to the severity of glaucoma^28–31^. Therefore, targeting the distal outflow pathway can be a viable mode of treatment for ocular hypertensive pathologies like glaucoma that involve perturbations of outflow resistance. However, the distal outflow pathway has not been studied in detail, mostly due to lack of model systems.

Given the importance of the distal outflow pathway in regulating IOP, understanding the biology and functional characteristics of this area is of great clinical relevance. Unfortunately, unlike primary NTM and SC cells, there are no established in vitro cell-based model systems that can be used to study the distal outflow pathway. To address this unmet need, we have isolated and characterized a specific population of vascular endothelial cells that retain and simulate the characteristics of the vessels of the distal outflow pathway. These vascular distal outflow pathway (VDOP) cells are phenotypically and functionally distinct from NTM and SC cells and represent an in vitro model system for understanding the biology of the distal outflow pathway of human eyes.

## Results

### Culture and proliferation of VDOP cells

**Figure 1:**
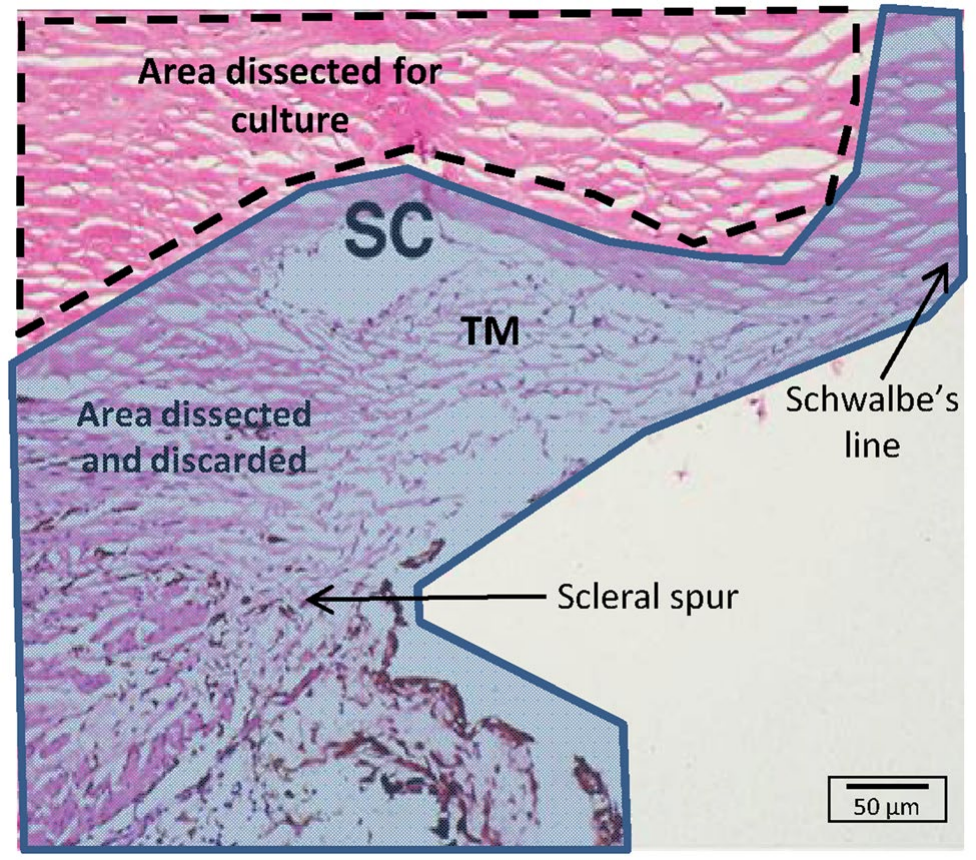
Establishing primary cultures of VDOP cells. Image showing the region of the conventional outflow pathway that was dissected and removed (lightly shaded portions), and the portion isolated (dotted area) for extracting VDOP cells. The extracted tissue rims were cut into 1–2 mm pieces, incubated in collagenase for 90 min and immersed in low glucose DMEM containing 10% FBS and 1% antibiotic/antimycotic on a gelatin-coated plate. Scale bar, 50 µm; SC, Schlemm’s canal; TM, trabecular meshwork.

**Figure 2:**
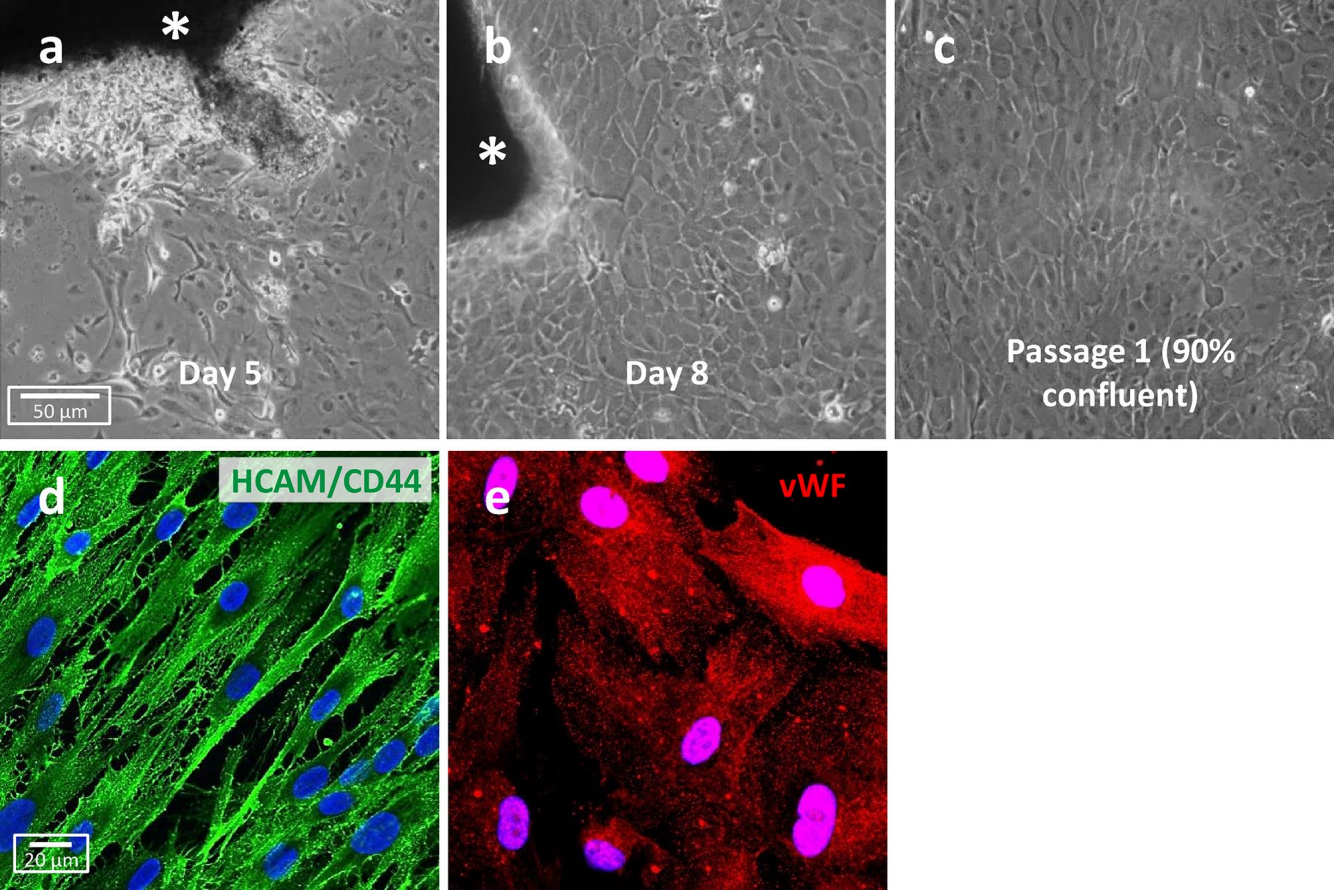
VDOP primary cells. (**a**, **b**) Gross morphological characteristics of VDOP cells growing out of tissue removed from the distal outflow region (asterisks). (**c**) Primary cell line of VDOP cells at 90% confluence. (**d**) VDOP cells stain positive for HCAM/CD44, highlighting the intercellular processes. (**e**) VDOP cells also show strong localization of von Willebrand factor (vWF), indicating a possible endothelial lineage of the cells. Representative images from VDOP cells at passage 4. Scale bar, 50 µm (**a**–**c**); 20 µm (**d**, **e**).

Demographics of the human donor eyes used to obtain primary cells are found in Table 1. To isolate VDOP cells from the distal outflow pathway of human anterior segments, the TM was removed after making vertical cuts anterior to Schwalbe’s line and posterior to the scleral spur and above the outer wall of SC, as previously described^13^. Once the TM was removed, an iris scalpel was used to repeatedly scrape and remove any remaining TM tissue as well as the inner and outer layer of SC. Cells were also scraped from the outside of the eye directly above the episcleral vasculature. Tissue distal to SC was isolated, treated with collagenase, and placed in gelatin-coated 6 well plates and incubated with Dulbecco’s modified eagle’s media (DMEM; Fig. 1). Cells migrated from the tissue (Fig. 2a,b) to the plate surface and reached 80–90% confluence within 8–10 days. Cells were transferred to a T75 tissue culture flask at seeding densities of 10^5^ cells and referred to as passage 1. Cells isolated and established from 8 independent human anterior segments showed a consistent doubling time of 48–72 h over passages 1–7. Proliferative potential beyond passage 7 was not evaluated.

**Table 1:** Demographic information of human donors of VDOP, NTM and SC primary cell lines.

VDOP cell line numbers	Age	Gender	Race
VDOP2	74	Female	Caucasian
VDOP3	88	Female	Caucasian
VDOP4	52	Male	American Indian/Alaskan native
VDOP5	82	Male	Caucasian
VDOP7	35	Male	Caucasian
VDOP8	20	Male	Undisclosed
VDOP9	50	Male	Caucasian
VDOP10	7	Female	Undisclosed
**NTM cell line numbers**			
NTM7	2	Female	Undisclosed
NTM14	32	Female	Undisclosed
NTM15	24	Male	Undisclosed
**SC cell line numbers**			
SC76	59	Female	Undisclosed
SC78	77	Male	Caucasian
SC89	68	Male	Caucasian

The overall morphology of VDOP cells exhibiting a distinct flat and cobblestone morphology is consistent with endothelial cells (Fig. 2c). VDOP cells show multiple interlinking processes that can be immunohistochemically stained with antibodies against homing cell adhesion molecule-1 (HCAM-1/CD44) (Fig. 2d). VDOP cells also stained positive for von Willebrand factor, further indicating the endothelial nature of the cells (Fig. 2e).

### Phenotypic characterization of VDOP cells

**Figure 3:**
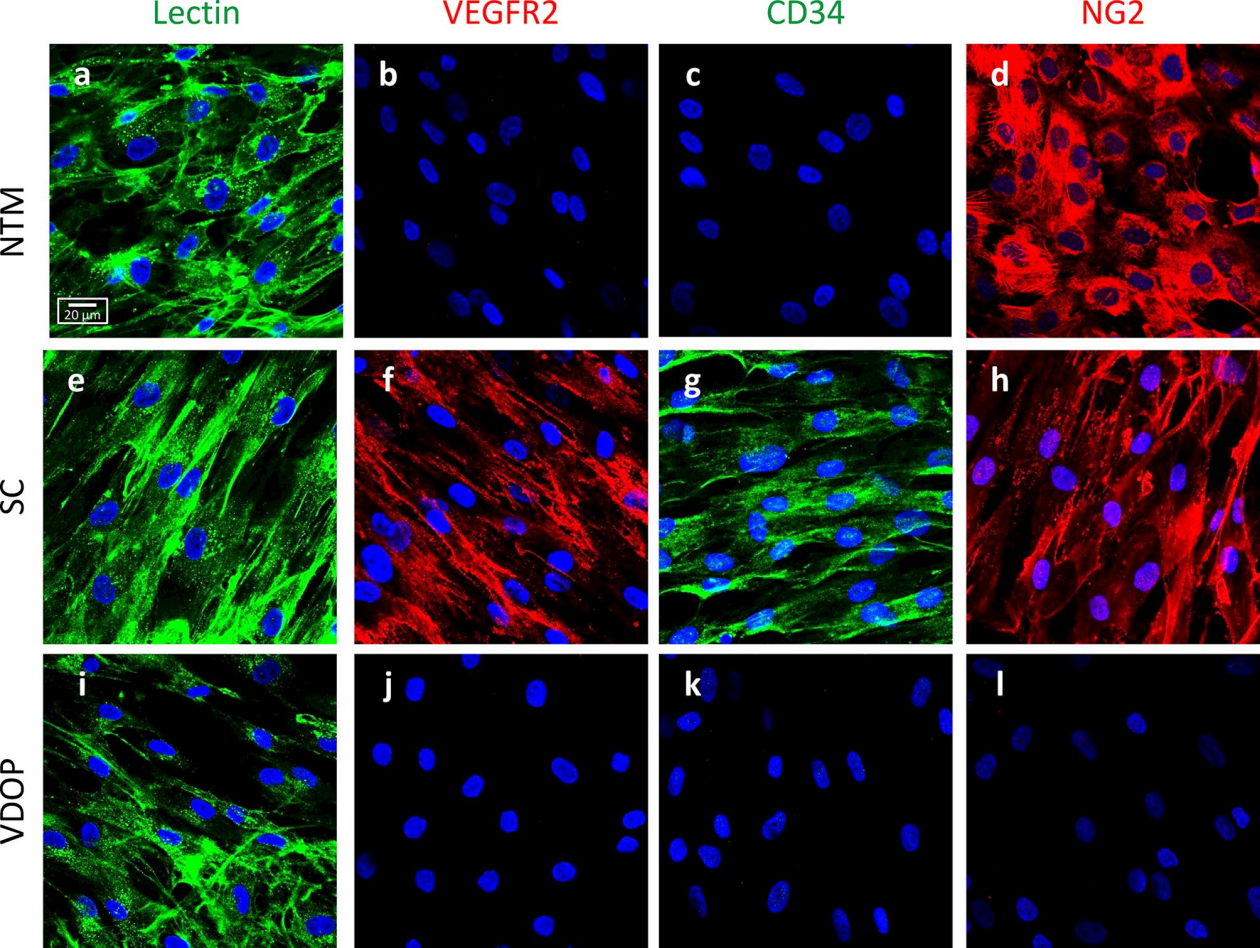
Phenotypic characterization of VDOP cells. Localization of various endothelial markers lectin, VEGFR2, CD34 and NG2 in NTM (**a**–**d**), SC (**e**–**h**) and VDOP (**i**–**l**) cells using immunohistochemistry. All three cell types were positive for Lectin (**a**, **e**, **i**) while VEGFR2 was seen only in SC and VDOP cells (**f**, **j**). CD34 was found only in SC cells (**g**) whereas both NTM and SC cells stained positive for NG2 (**d**, **h**). Based on this staining pattern, NTM cells were defined as Lectin^+^VEGFR2^-^CD34^-^NG2^+^, SC cells as Lectin^+^VEGFR2^+^CD34^+^NG2^+^ and VDOP cells as Lectin^+^VEGFR2^-^CD34^-^NG2^-^.A total of 8 VDOP (6 at passage 4 and 2 at passage 5), 3 NTM (2 at passage 5, one at passage 6) and 3 SC (all at passage 6) cell lines were used for this experiment. Scale bar, 20 µm.

To further evaluate the phenotypic characteristics of VDOP cells (n = 8 independent cell lines, 6 at passage 4, one at passage 5 and one at passage 8), immunohistochemical localization of a panel of endothelial cell surface markers (Table 2) were examined and compared to NTM (n = 3, 2 at passage 5, one at passage 6) and SC cells (n = 3, passage 6) to rule out contamination of nearby cells. All cell lines (VDOP, NTM and SC) stained positive for lectin, von Willebrand factor (vWF) and α-smooth muscle actin (α-SMA). Two out of the three SC cell lines tested positive for CD31 (PECAM1) while only one out of 8 VDOP cell lines showed CD31. CD34 was found in SC cells (2 out of 3 lines) but was not present in VDOP or NTMs. When stained for neuronal glial cell 2 (NG2), both NTM and SC cells showed more prevalent NG2 staining compared to VDOP cells (2 out of 3 NTM lines; 3 out of 3 SC lines; 1 out of 8 VDOP lines). However, VDOPs and NTMs were found to be less responsive to VEGFR2 staining (VDOP, 2 out of 8 lines; NTM, 1 out of 3 lines) compared to SC cells (all three lines) (Table 2). Based on percent of independent cell lines that tested positive or negative for any given markers (Table 2), VDOP cells displayed a distinguishing cell marker profile of Lectin^+^VEGFR2^-^CD34^-^NG2^-^ which was different from those shown by NTM (Lectin^+^VEGFR2^-^CD34^-^NG2^+^) and SC cells (Lectin^+^VEGFR2^+^CD34^+^NG2^+^) (Fig. 3).

**Table 2:** Various phenotypic markers and their staining frequencies in VDOP, NTM and SC cell lines.

Marker	Marker type	% Positive cells lines		
		VDOP (n = 8)	NTM (n = 3)	SC (n = 3)
Lectin	Endothelial	100	100	100
von Willebrand factor (vWF)	Microvessel	100	100	100
α-smooth muscle actin (α-SMA)	Myofibroblasts	100	100	100
CD31 (Platelet endothelial cell adhesion molecule, PECAM1)	Microvessel	12.5	0	67
CD34	Stem cell	0	0	67
Neuron-glial antigen 2 (NG-2)	Neuronal glial cell-2	12.5	67	100
Vascular endothelial growth factor-2 (VEGFR2)	Endothelial	25	33	100

**Figure 4:**
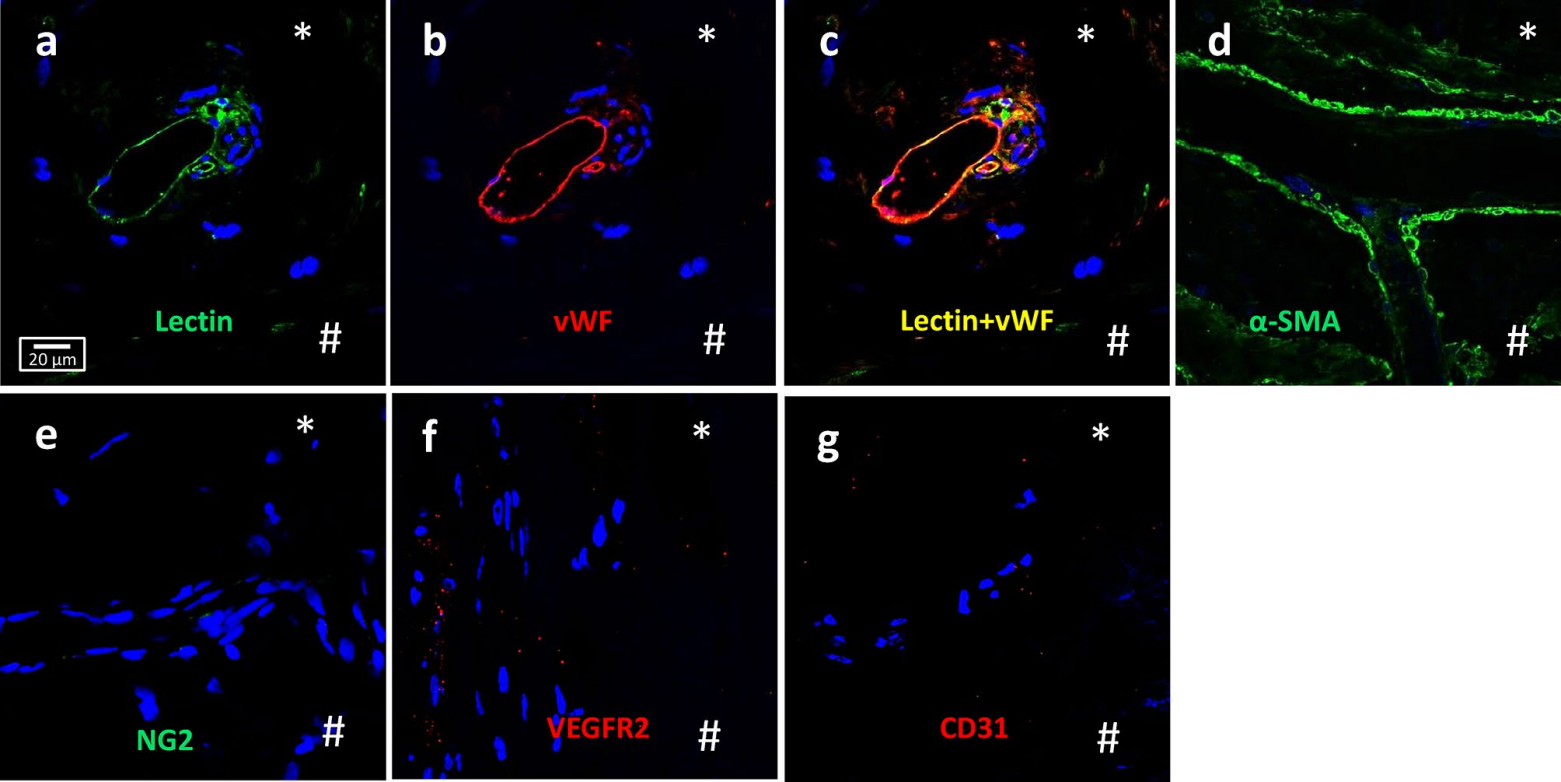
Phenotype characterization of micro-vessels in distal outflow region of human eyes. Representative images showing localization of lectin (**a**) and vWF (**b**) in micro-vessels found in paraffin sections of the distal outflow region (50–100 µm immediately downstream of outer wall of Schlemm’s canal and between the outer wall of Schlemm’s canal and episcleral region) of the conventional outflow pathway. When superimposed (**c**), the two molecules showed extensive co-localization in the endothelial cells of the micro-vessel. (**d**) Vessels from the distal outflow region were also positive for α-SMA but negative for NG2 (**e**), VEGFR2 (**f**) and CD31 (**g**). *Region of the section closest to the episcleral region; # area closest to the outer wall of Schlemm’s canal. Scale bar, 20 µm.

To determine if the VDOP cells retain and simulate their marker profile found in vivo, we evaluated localization of several positive markers identified on VDOP cells (lectin, α-SMA, vWF), in episcleral vessels identified in sections of the distal outflow region from two independent human donor eyes. Lectin and vWF co-localized in the endothelial cells of the vessels (Fig. 4a–c) and cells surrounding episcleral vessels also stained prominently for α-SMA (Fig. 4d). NG2, VEGFR2 and CD31, which were negative on VDOP cells, were similarly undetectable in the episcleral veins from ocular tissue sections (Fig. 4e–g). These results indicate that the isolated VDOP cells could be true representatives of endothelial cells of the distal outflow region.

### Physiological characteristics of VDOP cells

**Figure 5:**
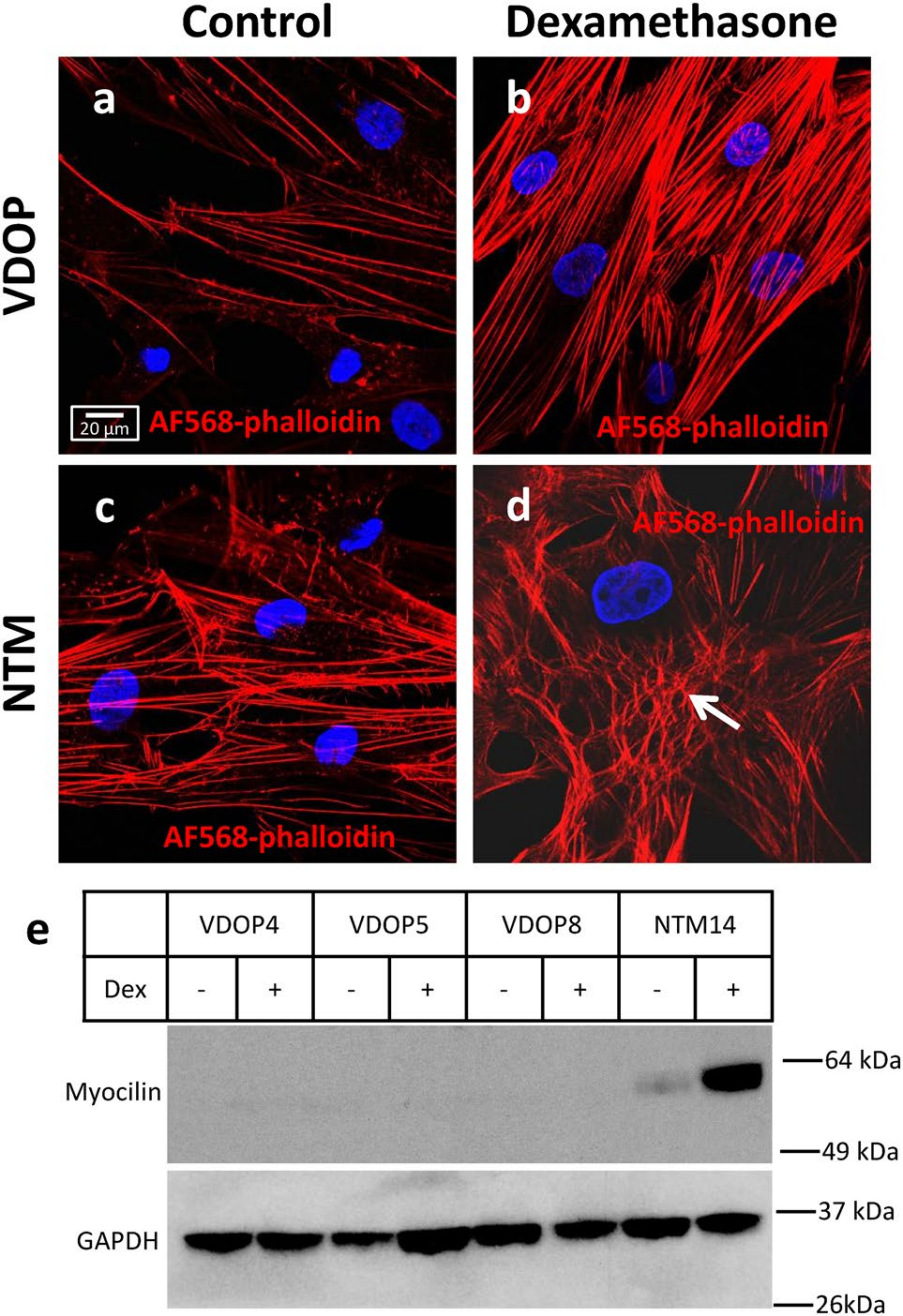
CLAN formation in NTM but not VDOP cell lines. (**a**, **b**) Representative confocal images showing complete absence of CLANs in VDOP cells after treatment with dexamethasone for 48 h. (**c**, **d**) NTM cells show extensive CLAN formation after similar treatment with dexamethasone (arrow). CLANs were identified by staining the actin cytoskeleton with Alexa Fluor 568 (red) conjugated phalloidin. (**e**) Expression of myocilin was not detectable in VDOP cells. However human NTM cells showed myocilin expression and upregulation following dexamethasone treatment. GAPDH was used as internal loading control. Full-length blots of myocilin and GAPDH are presented in supplementary Fig. 1a and 1b. Scale bar, 20 µm.

Previous studies have shown that primary NTM cells upregulate myocilin expression and form cross linked actin networks (CLANs) following dexamethasone treatment—physiologic parameters that are unique to these cells^8–10^. Following treatment with dexamethasone (10^–7^ M, 48 h), VDOP cells (n = 5) did not show any CLAN formation (Fig. 5a,b) whereas extensive CLANs were noted in NTM cells (n = 2) similarly treated with dexamethasone and used as positive controls (Fig. 5c,d). Western blot analysis of cell lysates isolated from VDOP and NTM primary cell cultures following treatment with dexamethasone (10^-7^ M, 48 h) showed no myocilin protein expression in VDOP cells, in contrast to NTM cells which show myocilin expression and upregulation following treatment (Fig. 5e). These data suggest that there are functional and phenotypic differences between VDOP and NTM primary cell cultures.

### Degree of similarity in independent VDOP cell lines isolated from different patients

**Figure 6:**
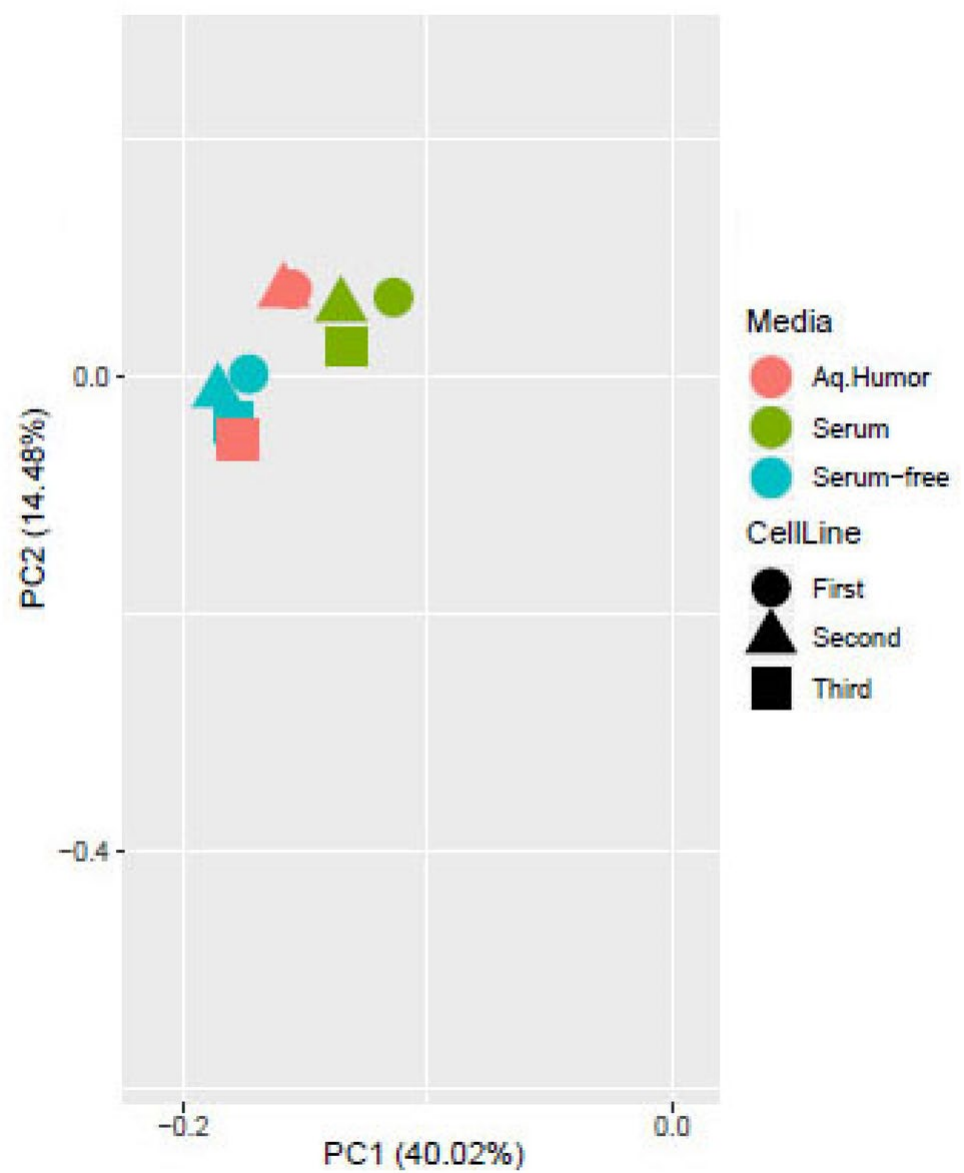
Comparison of independent VDOP cell lines in different media conditions. RNASeq analysis of total RNA from three independent VDOP lines (passage 5), grown in 50% human aqueous humor, 10% FBS and serum-free media. Principal component analysis shows closely localized clusters of all 3 cell lines in each media condition indicating high similarity between transcriptomes of the three independent VDOP cell lines.

Due to the primary nature of VDOP cell lines, they need to be repeatedly generated from independent donor eyes post mortem, to maintain a steady supply for experimental procedures. We evaluated whether our standardized technique for isolating these cells is able to select the same kind of cells from various patient samples. We analyzed the transcriptome from three independent VDOP cell lines, each grown in three different culture conditions (50% human aqueous humor, 10% fetal bovine serum (FBS), serum-free media) using RNASeq. Principal component analysis of the transcriptome from three cell lines, when reduced to a two dimensional plot, showed a single cluster for all cell lines across all culture conditions (Fig. 6). The high consistency in expressed transcription profiles across cell lines suggests that extraction of VDOP cells from the distal outflow region is consistent between isolates from independent donors.

## Discussion

With the distal outflow pathway gaining importance as a key component of aqueous humor outflow regulation in human eyes, there has been renewed interest in studying this region with the goal of identifying novel therapeutic targets that can be exploited to better treat ocular hypertensive diseases like glaucoma^16^. A major limitation to this strategy has been the lack of appropriate in vitro, cell culture based model systems for this region. Consequently, mechanistic aspects of the region’s involvement in outflow regulation are largely unknown. In the current study, we describe the successful isolation and culture of human vascular endothelial-like cells from the distal outflow region of the conventional outflow pathway. These VDOP cells are phenotypically and physiologically distinct from nearby TM and SC cells.

The method for isolating VDOP cells is highly reproducible, with a distinct characteristic marker profile of Lectin^+^VEGFR2^-^CD34^-^NG2^-^. This signature profile was used to validate each new cell line isolated from independent donor eyes. Additionally, RNASeq studies confirmed that independent VDOP primary cell lines (n = 3) had similar transcriptome profiles to each other when cultured in 50% human aqueous humor, 10% FBS or in serum-free media. These studies indicate that the initial dissection, cell separation and subsequent cell culture procedures consistently isolate and propagate a unique endothelial-like cell population from the distal outflow region. Due to the primary nature of the VDOP cells, mostly early passage cells (6 of the 8 VDOP lines were used at passage 4, one at passage 5 and one at passage 8) were used to investigate the marker phenotypes with only one cell line used at passage 8 to evaluate markers in a late passage. Although all cell lines showed similar results, to further validate the markers, we randomly selected two of the early passage VDOP cell lines and probed for select markers at passage 6 and found similar results as reported for passage 4 cells (data not shown). However, this study did not specifically look at the changes in marker profile across passage numbers. Our data only shows that primary cultures of VDOP cells up to passage 8 may be distinguished by the reported marker profile of Lectin^+^VEGFR2^-^CD34^-^NG2^-^.

Detailed analysis of the characteristics of VDOP cells, isolated from the distal outflow region indicates that they are functionally different from NTM cells. VDOP cells show neither dexamethasone-mediated upregulation of myocilin nor CLAN formation, both physiological responses seen in NTM cells^10,13,32^. These data suggest that VDOP cells are a unique cell population distinct from NTM cells. However, VDOP cells do share several common endothelial markers with NTM (viz. Lectin, vWF, α-SMA). This is not surprising, since cells of the TM are derived from the mesenchyme/neural crest and are endothelial in nature^33^. The endothelial glycoprotein marker lectin has been previously shown to be an important molecule in development of the anterior chamber angle and the TM^34^. However, unlike NTM cells, VDOP cells did not show expression of NG2, a pericyte/smooth muscle cell marker. Based on reported contractile properties of cells in the TM^29^, it is logical that we would see a smooth muscle marker on NTM cells. Overall, the phenotypic marker profiles show that while both NTM and VDOP cells share some common endothelial properties, they are distinct sub classes of endothelial cells.

SC cells originate from the mesodermal germ layer by a unique sequence of vascular development named canalogenesis^35^ and retain their vascular nature even in adult subjects^33,36^. Because of this, SC cells show several prominent endothelial markers (e.g. VEGFR2, CD34, CD31)^14,33,37^. Therefore, it was not surprising to see common endothelial markers such as lectin and vWF between VDOP and SC cells. However, VDOP cells did not show VEGFR2 or CD34, two markers that have been previously shown to be expressed in SC cells^33,35^. Their absence in VDOP cells further indicate that these unique cells are a sub-population of endothelial cells and distinct from SC cells. While VEGFR2 and CD34 are common endothelial/hematopoietic markers, they are expressed more in endothelial cells with strong potential for growth and restructuring and less in more established vascular beds like that of the episcleral plexus^35,38,39^.

Our primary goal for creating the VDOP cell lines was to develop a reproducible in vitro model for studying the involvement of the distal outflow region in regulation of aqueous outflow. While some authors have differentiated this area into deep scleral, intracscleral and episcleral plexus^20^, it is important to note that it was not possible to separate these through dissection. However, cells making up the endothelial-like vessels across the sub regions are similar in nature and the major difference between these regions appears to be in the proximity and complexity of the vascular beds^40,41^. Since VEGFR2, CD34 and NG2 are negative in VDOP cells, we could not use these to validate their presence in vivo in the vessels. For this reason we selected two separate markers viz. αSMA and vWF that were positive in VDOP cells and with lectin, showed that we could indeed find cells that were positive for all three (i.e. αSMA^+^vWF^+^Lectin^+^) in the vessels of the distal outflow pathway in vivo. The presence of lectin, vWF and α-SMA in both VDOP cells and tissue sections of the distal outflow vessels indicate that the cultured VDOP cells retain the vascular phenotype of their parent tissues and are representative of the distal outflow region.

While we have carefully distinguished VDOP cells from NTM and SC cells, we cannot rule out the possibility that our cultures contained cells from other nearby tissues. The most likely cellular contaminants would be scleral or conjunctival cells. However, scleral cells are mostly terminally differentiated keratinized cells and it is unlikely that these cells would de-differentiate and show proliferative properties similar to what we see in VDOP cells. To prevent entry and growth of conjunctival cells in our cultures, we removed the conjunctiva from the donor eyes, followed by vigorous scraping of the sclera with a scalpel to remove conjunctival remnants prior to dissection. Given that conjunctival cells are more epithelial in nature^42^ whereas VDOP cells are endothelial-like, it seems unlikely that VDOP cells are derived from contamination of conjunctival cells. Furthermore, with RNASeq profiles that are nearly identical between different VDOP cell lines, the possibility of a significant contamination is highly unlikely.

There are numerous avenues of research where VDOP cell lines could be useful. For example, evaluating if and how these cells are altered during disease could significantly aid in understanding mechanistic events during ocular hypertensive disorders like glaucoma. VDOP cells may be a valuable in *vitro* model system to study how current and future ocular hypotensive drugs or other general agents (small molecules, biological compounds, etc.) can and will affect the physiological function of these cells. Additionally, experiments in primary VDOP cells can aid in designing experiments in animal models. With the use of the described methodology, VDOP cells can be utilized as an in vitro model system for researchers to investigate the cell and molecular mechanisms involved in normal and disease processes in the distal outflow region.

## Methods

### Isolation and cell culture conditions for VDOP, NTM and SC cells

All human tissues were used as per guidelines of the Declaration of Helsinki and the study was pre-approved by Mayo Clinic Institutional Review Board. Demographic information of human donors used for isolation of VDOP, NTM and SC primary cell lines are provided in Table 1.

#### VDOP cells

A total of 8 pairs of corneo-scleral rims were used for this study. All eye tissues were free from ocular disorders and malignancies and were supplied by the Lions Gift of Sight (St. Paul, MN) under our pre-approved IRB protocol. Informed consent was obtained from the family of the deceased by the Lions Gift of Sight, prior to tissue collection. The tissues were obtained without any patient identifiers, except for the age, gender and general cause of death. Corneo-scleral rims were stored while immersed in Optisol (Numedi, Isanti, MN) at 4 °C. All tissues were processed for cell isolation within 48–72 h of death. During cell isolation, corneo-scleral rims were taken out of optisol and washed 3 times with PBS while gently shaking for 1 min between PBS exchanges. Vertical cuts were made anterior to Schwalbe’s line and posterior to the scleral spur and the entire TM tissue was removed under a dissecting microscope (SZX16, Olympus, Tokyo, Japan). The area under the TM was vigorously scraped to remove any TM remnants as well as to remove the outer wall of SC. The dorsal surface of the sclera (opposite the TM) was similarly scraped to remove conjunctival cells and the epithelial cells from the top layer of the sclera. Using a curved scissor, a small ring was cut out around the entire corneal rim (minus the cornea) that included the distal portion of the conventional outflow pathway, downstream from SC (Fig. 1; Supplementary Fig. 1). It should be noted that some authors have further characterized the distal portion of the conventional outflow pathway as deep scleral plexus (immediately distal to SC), intracscleral plexus (anterior to deep scleral plexus) and episcleral plexus (region closest to the dorsal portion of the sclera) ^20,40,41^. However, it was not technically feasible to manually separate these areas and therefore the entire distal region was utilized for extraction of VDOP cells.

The dissected ring containing the distal region of the conventional outflow pathway was cut into 1–2 mm pieces and rinsed twice in Hank’s balanced salt solution (HBSS; with Ca^2+^ and Mg^2+^; Mediatech, Manassas, VA). These 1–2 mm tissue pieces from right and left eyes were combined and incubated in collagenase (100U/ml in HBSS with Ca^2+^Mg^2+^) (Mediatech) for 90 min and washed again with HBSS without collagenase (3 × 5 min). After the last wash, tissues were centrifuged and distributed to 2 wells of a gelatin coated 6 well tissue culture plate (Biocoat, Corning, Kennebunk, ME) using a wide bore plastic transfer pipette. A sterile 22 × 22 mm cover slip (Fisher Scientific, Waltham, MA) was placed on top of the tissue pieces to prevent them from dislodging. The cover slip was submerged under 1 ml of low glucose DMEM (Gibco, Grand Island, NY) containing 10% FBS (Gibco) and 1% antibiotic/antimycotic (Sigma-Aldrich, St. Louis, MO) solution. The plate was placed in a humidified cell culture incubator at 37 °C and 5% CO_2_.

Cellular strands extending from tissue pieces would appear within 3–5 days of culture initiation. At 8–10 days, media was removed and cells were washed 3 times with PBS. Trypsin–EDTA (ThermoFisher Scientific, Waltham, MA) was added to each well and plates were incubated at 37 °C for 3–5 min. This allowed most of the tissues as well as the cells to detach from the plate. The remaining tissue pieces were manually removed by gentle scraping. Tissue pieces and trypsinized cells were transferred to a 15 ml conical tube and washed with PBS twice and centrifuged. The cell pellet was resuspended in DMEM containing 10% FBS and 1% antibiotic/antimycotic, and transferred to a single well of a 6 well tissue culture plate (without gelatin coating). Floating tissue pieces were manually removed with forceps and cells were allowed to settle and proliferate. This was considered passage 0. Once cells reached 90% confluence (3–5 × 10^5^ cells), they were trypsinized, pelleted by centrifugation, resuspended in DMEM containing 10% FBS and transferred to a T75 cell culture flask (Corning, Corning, NY). VDOP cells were subsequently split at 1:4 ratios at 90% confluence. Doubling time was calculated based on the formula [Doubling time = {duration*log(2)}/{log(final concentration) – log(initial concentration)}]. For subsequent experiments, cells beyond passage 5 were not used in functional studies, although the cells were observed to maintain consistent proliferative potential up to passage 7. Growth properties beyond passage 7 were not evaluated.

#### NTM cells

Primary cultures of NTM cells (n = 3) were established according to previously reported protocols^43,44^. Cells were maintained in low glucose DMEM (Gibco) containing 10% FBS (Gibco) and 1% antibiotic/antimycotic (Sigma-Aldrich) in a humidified incubator at 37 °C with 5% CO_2_ and split at 1:4 ratios. All experiments were performed in cells that were cultured for ≤ 7 passages.

#### SC cells

SC cell lines (n = 3) were generous gifts from Dr. W. Daniel Stamer (Duke University, Durham, NC). Procedures for establishing primary cultures of SC cells and their characterization have been previously described^14^. Similar to VDOP and NTM cells, SC cells were grown in low glucose DMEM (Gibco) containing 10% FBS (Gibco) and 1% antibiotic/antimycotic (Sigma-Aldrich) and passaged at 1:3 ratios. Only cells ≤ 7 were used for experiments.

### Evaluation of VDOP phenotype using immunohistochemistry (IHC)

VDOP, NTM and SC cells were grown to confluence in 8 well chamber slides (Millicell EZ slide, Millipore, Tullagreen, Ireland), fixed in 4% paraformaldehyde, blocked in 1% bovine serum albumin (Sigma-Aldrich, St. Louis, MO), and incubated with a panel of primary antibodies (Table 3) overnight. Primary antibodies were removed, chambers washed with PBS (3 × 5 min), and incubated with appropriate fluorophore conjugated secondary antibodies (Table 3). Following washes in PBS, chamber partitions were removed and slides were mounted under Vectashield mounting medium containing DAPI (Vector Laboratories, Burlingame, CA). Cells were examined under a confocal laser microscope (Zeiss LSM 780; Carl Zeiss, Thornwood, NY) and laser intensity was adjusted based on negative controls where only secondary antibody was added. Cell lines were considered positive for any given marker when > 80% of the cells stained positive. Out of the 8 VDOP cell lines used for IHC experiments, 6 were used at passage 4, one at passage 5 and one at passage 8.

**Table 3:** List of antibodies and their distribution source.

Primary antibodies	Secondary antibodies
FITC-conjugated Lectin, Sigma-Aldrich (St. Louis, MO) Cat. No. L0401	NA
Rabbit polyclonal, vWF Abcam (Cambridge, MA) Cat. No. ab6994	Alexa Fluor (AF) 546 conjugated anti-rabbit, Invitrogen (Carlsbad, CA) Cat. No. A11010
Mouse monoclonal α-SMA, Sigma-Aldrich Cat. No. A2547	AF 488 conjugated anti-mouse, Invitrogen Cat. No. A11059
Mouse monoclonal CD31, R&D Systems (Minneapolis, MN) Cat. No. BBA7	AF 488 conjugated anti-mouse, Invitrogen Cat. No. A11059
Mouse monoclonal CD34, Cell Signaling Technology (Danvers, MA) Cat. No. 07/2017	AF 488 conjugated anti-mouse, Invitrogen Cat. No. A11059
Mouse monoclonal NG-2, Millipore (Billerica, MA) Cat. No. 05–710	AF 546 conjugated anti-mouse, Life Technologies (Carlsbad, CA) Cat. No. A11003
Rabbit monoclonal VEGFR2, Cell Signaling Technology Cat. No. 08/2017	AF 546 conjugated anti-rabbit, invitrogen Cat. No. A11010
Rat monoclonal HCAM, Santa Cruz Biotechnology (Dallas, TX) Cat. No. sc-18849	AF488 conjugated anti –rat, Invitrogen Cat. No. A11006

For tissue staining, the distal outflow pathway region was dissected (as described above), fixed in 10% neutral buffered formalin (Fisher Scientific), dehydrated in increasing ethanol concentrations (75%, 85%, 95% and 100%) and embedded in paraffin. Paraffin blocks were cut into 5 µm sections, mounted on Superfrost/plus glass slides (Fisher Scientific, Pittsburgh, PA) and incubated at 60 °C for 2 h. Prior to immunohistochemistry, tissue sections were soaked in xylene to remove paraffin and then rehydrated in decreasing ethanol concentrations (95%, 85%, 75%). Antigen retrieval was performed by incubating slides in 1 mM EDTA (pH 8.0) at 95 °C for 30 minutes^45^. Sections were blocked and permeabilized in PBS containing 3% BSA and 0.1% TritonX-100, probed with lectin, vWF, and αSMA primary and corresponding secondary antibodies (Table 3), mounted in Vectashield containg DAPI (Vector Laboratories) and imaged by confocal microscopy (described above).

### Evaluation of CLAN formation following dexamethasone treatment

CLANs were evaluated by immunohistochemical methods as described above. Briefly, cells were grown to confluence in 8 well chamber slides (Millicell EZ slide, Millipore). The day prior to initiation of experiment, cells were placed in serum-free media. The next day, cells were treated with either 10^-7^ M dexamethasone dissolved in 2% methanol in serum-free DMEM or vehicle alone for 48 h. Fresh dexamethasone was added after 24 h. Following treatment, cells were washed and incubated for 15 min with Alexa Fluor 568 conjugated phalloidin (Invitrogen, Carlsbad, CA). After final washes in PBS, chamber partitions were removed, cells were mounted in Vectashield containing DAPI (Vector Laboratories), and imaged with a Zeiss LSM 780 confocal microscope (Carl Zeiss). CLANs were identified as geodesic dome like structures engaging either part or whole of the cellular cytoskeleton^9,10,46^ under water corrected 63X lens.

### Western blot analysis of myocilin expression following dexamethasone treatment

NTM and VDOP cells were grown to confluence in 6 well plates. Evening prior to study, cells were washed with PBS and incubated in serum-free media overnight. The following morning, cells were treated with 10^–7^ M dexamethasone for 48 h with fresh dexamethasone being added for a second time after the first 24 h. Cells were lysed directly in the wells using an ice-cold lysis buffer (50 mM Tris, pH 8.0, 0.5% sodium dodecyl sulfate, 0.5% Triton X-100, 137 mM NaCl, 3 mM KCl, 8 mM Na_2_HPO_4_-7H_2_O, 1 mM KH_2_PO_4_) pre mixed with protease and phosphatase inhibitors (Roche, Indianapolis, IN), collected and stored at − 80 °C until further use.

After analyzing total protein content using Bradford’s assay, cell lysates were mixed with 5X reducing sample buffer (Thermo Scientific, Waltham, MA) containing 15% β-mercaptoethanol (Sigma-Aldrich). Equal amounts of total protein (15–20 µg) was loaded in each well of a 4–15% gradient SDS-PAGE gel (Bio-Rad, Hercules, CA), transferred to polyvinylidene difluoride membranes (Millipore, Billerica, MA) and blocked in 2% non-fat dried milk, as described previously^43,47^. Once protein transfer was complete, blots were probed with primary antibodies against rabbit anti-human polyclonal myocilin (developed in our laboratory against the peptide sequence ARPQETQEGLQRELGTLRRERDQLC)^48^ overnight. Following incubation, blots were washed with Tris-buffered saline containing Tween 20 (20 mM Tris [pH 7.5], 150 mM NaCl, 0.05% Tween-20) and incubated with horse-radish peroxidase conjugated anti-rabbit secondary antibody (GE Healthcare, Piscataway, NJ). Protein bands were visualized with ECL western blot signal detection reagent (GE Healthcare) and Kodak Biomax XAR films (Eastman Kodak, Rochester, NY). After probing for myocilin, membranes were stripped with 5 M guanidine hydrochloride (Sigma-Aldrich), washed in deionized water and reprobed with mouse anti-human GAPDH (Novus Biologicals, Littleton, CO) which was utilized as loading control, followed by horse-radish peroxidase conjugated anti-mouse secondary antibody (GE Healthcare).

### RNA sequencing

Total RNA was isolated from three independent confluent VDOP cell lines using RNeasy mini kit (Qiagen, Valencia, CA) following the manufacturer’s protocol. An RNA library was prepared for each cell line using the TruSeq RNA sample Prep kit version 2 (Illumina, San Diego, CA, USA). In samples with RNA integrity number > 7.0, ribosomal transcripts were depleted and resulting libraries were minimally amplified and quantified for sequencing using a HiSeq4000 sequencer (Illumina, San Diego, CA) as previously described^49^. Transcriptomic sequencing data from each cell line was processed at AccuraScience LLC (Johnston, IA). Multidimensional sequencing data were visualized using principal component analysis charts.

## Supplementary Information

Supplementary Information 1.
