# Supplementary material for: Isolation and characterization of novel primary cells from the human distal outflow pathway
Source: Sci Rep. 2021 Feb 17;11:4034. doi: 10.1038/s41598-021-83558-6 (PMC7890058; doi:10.1038/s41598-021-83558-6)
Supplement: Supplementary file 1 — Supplementary Information 1. [file 41598_2021_83558_MOESM1_ESM.pdf]

Isolation and characterization of novel primary cells from the human distal outflow pathway

Uttio Roy Chowdhury, Cindy K. Bahler, Cheryl R. Hann, Bradley H. Holman, Michael P. Fautsch

Supplementary data

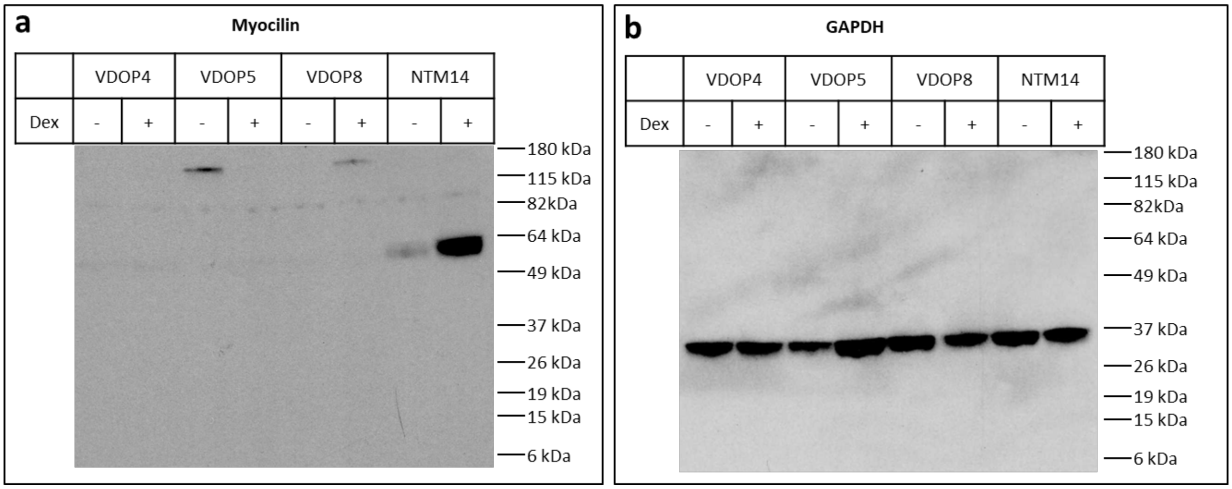

Supplementary figure 1: Full sized western blot images for myocilin (a) and GAPDH (b) as referred to in figure 5e of main manuscript.

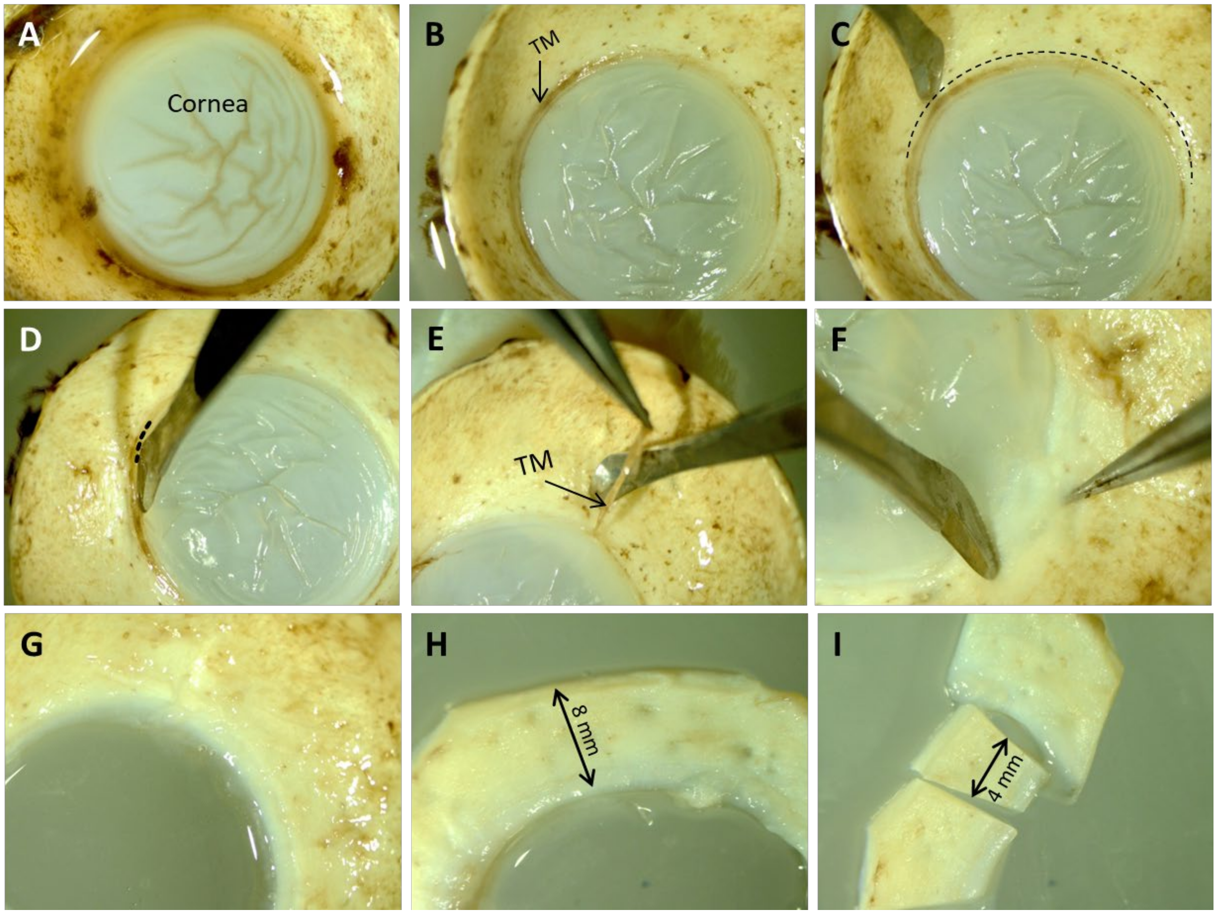

Supplementary figure 2. Stepwise demonstration of various dissection steps involved in isolation of the distal outflow region from which the VDOP cells are extracted.

A. The corneo scleral rim is dissected out. Lens, iris, ciliary body and retina are removed.

B. The rim is washed with PBS and gently scraped with a microsurgery knife on both sides to remove conjunctiva and remnants of ciliary body.

C. A microsurgery scalpel is used to make shallow cuts along the circumference above the outer wall of the Schlemm's canal (as shown by the dotted line).

D. A second shallow incision is made posterior to the scleral spur, along the dotted lines.

E. The trabecular meshwork (TM) is pulled out.

F. The area underneath the meshwork, including the Schlemm's canal outer wall, is vigorously scraped with the microsurgery scalpel.

G. The cornea is removed with a 10 mm corneal punch.

H. The excess scleral tissue is dissected out leaving only a thin ring of tissue 8-10 mm wide.

I. The ring is cut into small pieces, less than 5 mm wide, with a scalpel. These small tissue pieces are next incubated in collagenase as described in the methods section of the main manuscript.

For the purpose of this manuscript and in order to maintain clarity, these images were taken without immersing the tissues into any buffer. During actual extraction of the cells, tissues are dissected under PBS to maintain viability of cells. TM, trabecular meshwork.
